# Targeted therapy outcomes in acrodermatitis continua of Hallopeau: A systematic review

**DOI:** 10.7555/JBR.38.20240090

**Published:** 2024-08-20

**Authors:** ChaoJing Zhou, YiYun Hou, YuFei Wang, JiLiang Lu, YaMei Gao, ZhiQiang Yin

**Affiliations:** Department of Dermatology, the First Affiliated Hospital of Nanjing Medical University, Nanjing, Jiangsu 210029, China

Dear Editor,

Acrodermatitis continua of Hallopeau (ACH), a rare and chronic variant of pustular psoriasis, is characterized by atrophic skin changes, sterile pustules, onychodystrophy, and osteolysis of the distal phalanges of the fingers and toes. Given the absence of international consensus guidelines and comprehensive clinical studies of high methodological rigor, the management of ACH primarily relies on antipsoriatic therapeutic approaches and empirical evidence derived from scattered case reports. With the development of biologics and small-molecule drugs, targeted therapies hold some promise for the future of its successful management ^[ 1] ^. Here we summarize treatment outcomes of targeted therapies for ACH.

Based on the MEDLINE and Embase databases, we collected the published data of targeted therapies for ACH before March 5, 2024 ( ***Supplementary Table 1*** and ***Supplementary Fig. 1***, available online). Our review identified 268 records through the two electronic databases. In total, 66 records met the inclusion criteria, including 134 patients with 209 targeted treatments (mean age: 44.0 years, and male: 45.5%; ***Supplementary Table 2***, available online). The mean disease duration was 6.7 years, and 19.4% of patients had received the targeted therapy before. The treatments administered were interleukin-17 (IL-17) inhibitors (20.6%, *n* = 43/209), IL-12/23 inhibitors (18.2%, *n* = 38/209), tumor necrosis factor-α (TNF-α) inhibitors (51.2%, *n* = 107/209), IL-1 inhibitors (1.4%, *n* = 3/209), IL-36 inhibitors (0.5%, *n* = 1/209), anti-CD11a antibodies (1.4%, *n* = 3/209), phosphodiesterase 4 (PDE4) inhibitors (5.7%, *n* = 12/209), and Janus kinase (JAK) inhibitors (1.0%, *n* = 2/209; ***Supplementary Table 3***, available online) *.*

To collect additional solid evidence, we conducted a thorough analysis of 11 case series and cohort studies, including 79 patients with 106 targeted treatments. Information about patient details, clinical features, and prior failed treatments was collected ( ***Supplementary Table 4***, available online). Among these patient cases, 10 cases (12.7%) were identified as enthesitis and five cases (6.3%) showed bone destruction ( ***Supplementary Table 5***, available online). The treatment response was categorized into three types: (1) complete resolution (CR) that was defined by phrasing such as "marked response", "remission", "regression", "dramatic improvement", "almost complete clearance", or similar description found in medical records or photographic evidence; (2) partial resolution (PR) that was defined by phrasing such as "some improvement" or "limited relief" in medical records or photographic evidence; and (3) no resolution (NR) that was defined by phrasing such as "no response", "ineffectiveness", or exacerbation of the disease. The IL-12/23 inhibitor treatment showed the highest frequency of cases with improvement: CR in 52.6% ( *n* = 10/19) of the cases within 22.4 weeks. The TNF-α inhibitor treatment showed the second highest frequency of improvement: CR in 44.1% ( *n* = 26/59) of the cases within 14.6 weeks, and PR in 18.6% ( *n* = 11/59) of the cases, but with a slightly higher (15.3% [ *n* = 15/59]) incidence of adverse events. A minority of cases reported a rapid initial response to the TNF-α inhibitor treatment but showed a lack of sustained effectiveness over time. The IL-17 inhibitor treatment achieved CR in 26.1% ( *n* = 6/23) of the cases within 24.0 weeks ( ***Table 1***). Moreover, regardless of the type of biological agent used, the targeted treatment responses were generally more evident in female patients than in male patients. The highest excellent response rate was observed in female patients who were treated with IL-12/23 inhibitors (CR: 75.0%, *n* = 3/4; ***Supplementary Table 6***, available online).

**Table 1:** Summary of targeted therapy outcomes in patients with acrodermatitis continua of Hallopeau

Variables	IL-17 inhibitors ( *n* = 23)	IL-12/23 inhibitors ( *n* = 19)	TNF-α inhibitors ( *n* = 59)	IL-1 inhibitors ( *n* = 1)	CD11a antibodies ( *n* = 1)	PDE4 inhibitors ( *n* = 2)	JAK1/2 inhibitors ( *n* = 1)
Treatment [ *n* (%)]	Ixekizumab, 9 (39.1) Secukinumab, 13 (56.5) Brodalumab, 1 (4.3)	Ustekinumab, 13 (68.4) Guselkumab, 5 (26.3) Risankizumab, 1 (5.3)	Etanercept, 16 (27.1) Infliximab, 12 (20.3) Adalimumab, 31 (52.5)	Anakinra, 1 (100)	Efalizumab, 1 (100)	Apremilast, 2 (100)	Baricitinib, 1 (100)
Treatment class outcomes [ *n* (%)]	CR, 6 (26.1) PR, 3 (13.0) NO, 14 (60.9)	CR, 10 (52.6) PR, 2 (10.5) NO, 7 (36.8)	CR, 26 (44.1) PR, 11 (18.6) NO, 22 (37.3)	NO, 1 (100)	NO, 1 (100)	NO, 2 (100)	NO, 1 (100)
Complete resolution time (weeks)	24.0 ^a^	22.4 ^b^	14.6 ^c^	NA	NA	NA	NA
Follow-up period (months)	7.0 ^d^	26.2 ^e^	16.0 ^f^	NA	NA	NA	NA
Adverse events [ *n* (%)]	Yes, 2 (8.7) None, 7 (30.4) NR, 14 (60.9)	Yes, 2 (10.5) None, 10 (52.6) NR, 7 (36.8)	Yes, 9 (15.3) None, 22 (37.3) NR, 28 (47.4)	NA	NA	NA	NA
^a^The CR time recorded for three of the six cases achieved CR. ^b^The CR time recorded for five of the ten cases achieved CR. ^c^The CR time recorded for 14 of the 26 cases achieved CR. ^d^The follow-up period recorded for two of the 23 cases received IL-17 inhibitors therapy. ^e^The follow-up period recorded for five of the 19 cases received IL-12/23 inhibitors therapy. ^f^The follow-up period recorded for five of the 59 cases received TNF-α inhibitors therapy.Abbreviations: CR, complete resolution; PR, partial resolution; NO, no resolution; NR, not reported; NA, not available; IL, interleukin; TNF, tumor necrosis factor; PDE, phosphodiesterase; JAK, Janus kinases.							

Most therapies for ACH have been based on traditional treatment approaches for plaque psoriasis, such as cyclosporine, acitretin, methotrexate, and cyclosporine ^[ 2] ^. One study suggested that biologics might be of use to clinicians in managing ACH as a second-line treatment ^[ 3] ^. Nevertheless, the choice of treatment should always be tailored to individual patients, considering the extent and severity of their disease. As shown in our data, a high proportion of patients faced the risk of disability caused by anonychia and bone erosions. There remains a need for treatments with a rapid action as well as a high and sustained efficacy. Our data also suggested that IL-12/23 inhibitors, particularly guselkumab, were highly effective, and IL-17 inhibitors, such as ixekizumab and secukinumab, showed a favorable risk profile. Differences in treatment efficacies between sexes may be influenced by factors such as drug dosage ratios, medical conditions, and other variables. Meanwhile, because of the extreme rarity of the disorder, several new targeted therapies still lack sufficient data. Mechanistically, IL-36 cytokines are likely the pivotal driver of the autoinflammatory responses that characterize pustular psoriasis ^[ 4] ^. One case was a child with ACH, who was successfully treated with spesolimab ^[ 5] ^, indicating that IL-36 inhibitors may be a possibly promising therapeutic option. Furthermore, small molecule therapeutics, such as apremilast and baricitinib, have been reported to yield excellent treatment outcomes in several cases, which are potentially more convenient and cost-effective for the patients ^[ 6– 8] ^.

Limitations include a lack of evidence from randomized controlled trials, the absence of strict monotherapies, and small sample sizes. Treatment failures are usually not reported in the medical literature, which may influence the statistics of outcomes in terms of possible reporting bias. In conclusion, the targeted therapies are an effective modality for refractory ACH, particularly in cases where traditional methods have been proven inadequate.

The present work was supported by the National Natural Science Foundation of China (Grant Nos. 82073439 and 82373475).

Yours Sincerely,Chaojing Zhou ^△^, Yiyun Hou ^△^, Yufei Wang ^△^, Jiliang Lu, Yamei Gao, Zhiqiang Yin ^✉^ Department of Dermatology, the First Affiliated Hospital of Nanjing Medical University,Nanjing, Jiangsu 210029,China. ^△^These authors contributed equally to this work. ^✉^Corresponding author: Zhiqiang Yin. E-mail: yinzhiqiang@njmu.edu.cn.

## SUPPLEMENTARY DATA

Supplementary data to this article can be found online.
