# Supplementary material for: Targeted therapy outcomes in acrodermatitis continua of Hallopeau: A systematic review
Source: J Biomed Res. 2024 Aug 20;38(6):640–2. doi: 10.7555/JBR.38.20240090 (PMC11629156; doi:10.7555/JBR.38.20240090)
Supplement: Supplementary file 1 — Supplementary data to this article can be found online. [file jbr-38-6-640-S1.pdf]

Available online at www.jbr-pub.org.cn

Open Access at PubMed Central

Journal of Biomedical Research, 2024 38(6): 1–4

Supplementary Data

JBR

Targeted therapy outcomes in acrodermatitis continua of Hallopeau: A systematic review

Chaojing Zhou△, Yiyun Hou△, Yufei Wang△, Jiliang Lu, Yamei Gao, Zhiqiang Yin✉

Department of Dermatology, the First Affiliated Hospital of Nanjing Medical University, Nanjing, Jiangsu 210029, China.

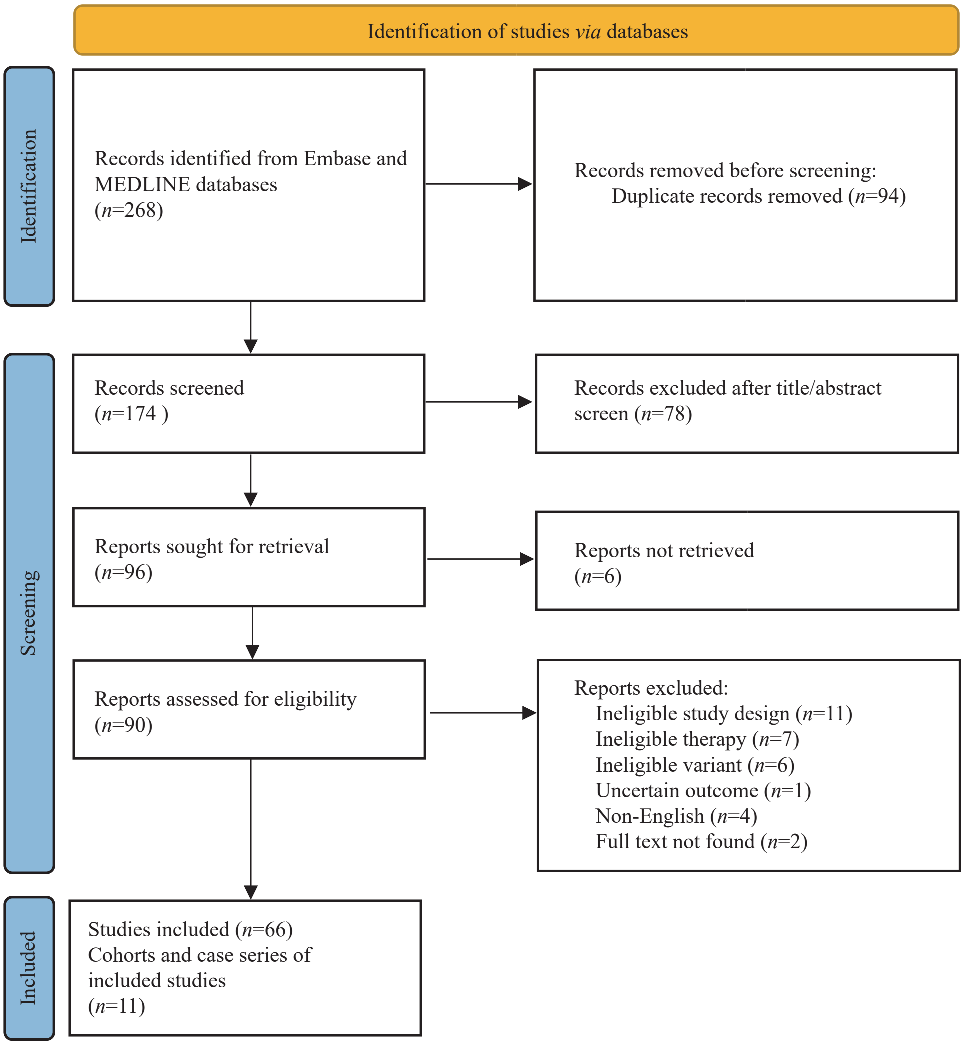

Supplementary Fig. 1 **Selection for the study inclusion.**

△These authors contributed equally to this work.

✉Corresponding author: ZhiQiang Yin, Department of Dermatology, the First Affiliated Hospital of Nanjing Medical University,Nanjing, Jiangsu 210029, China. E-mail: yinzhiqiang@njmu.edu.cn.

Received: 03 April 2024; Revised: 25 July 2024; Accepted: 29 July 2024; Published online: 20 August 2024

CLC number: R758.6, Document code: A

The authors reported no conflict of interests.

This is an open access article under the Creative Commons Attribution (CC BY 4.0) license, which permits others to distribute, remix, adapt and build upon this work, for commercial use, provided the original work is properly cited.

© B by Journal of Biomedical Research.

https://doi.org/10.7555/JBR.38.20240090

Supplementary Table 1 **Full search strategies in MEDLINE and Embase from inception through March 5, 2024**

Data bases	Strategies
MEDLINE	#1 (acrodermatitis continua) OR (Hallopeau)<br>#2 ((((((((((((TNF-α) OR (Etanercept)) OR (Enbrel)) OR (Erelzi)) OR (Infliximab))) OR (Remicade)) OR (Adalimumab)) OR (Humira)) OR (Amjevita)) OR (Certolizumab)) OR (Cimzia)) OR (Golimumab)) OR (Simponi)<br>#3 ((((((((IL 17) OR (Bimekizumab)) OR (Bimzelx)) OR (Ixekizumab)) OR (Taltz)) OR (Brodalumab)) OR (Siliq)) OR (Secukinumab)) OR (Cosentyx)<br>#4 (((((((((IL 23) OR (IL 12)) OR (Ustekinumab)) OR (Stelara)) OR (Guselkumab)) OR (Tremfya)) OR (Risankizumab)) OR (Skyrizi)) OR (Briakinumab)) OR (Siliq)<br>#5 ((IL-36) OR (Spesolimab)) OR (Spevigo)<br>#6 ((((((JAK inhibitor) OR (Tofacitinib)) OR (Xeljanz)) OR (Ruxolitinib)) OR (Baricitinib)) OR (BMS-986 165)) OR (Peficitinib)<br>#7 ((PDE 4) OR (Apremilast)) OR (Aprezo)<br>#8 (((((#2) OR (#3)) OR (#4) OR (#5)) OR (#6)) OR (#7))<br>#9 (#1) AND (#8)
Embase	#1 'acrodermatitis continua'/exp<br>#2 'acrodermatitis of hallopeau':ab,ti OR 'acrodermatitis perstans':ab,ti OR 'acrodermatitis pustulosa continua':ab,ti OR 'acrodermatitis pustulosa perstans':ab,ti OR 'acropustulosis':ab,ti OR 'dermatitis repens':ab,ti OR 'hallopeau acrodermatitis':ab,ti OR 'hallopeau disease':ab,ti OR 'hallopeau`s acrodermatitis':ab,ti OR 'hallopeau`s continuous acrodermatitis':ab,ti OR 'hallopeau`s disease':ab,ti OR 'pustular acrodermatitis':ab,ti OR 'acrodermatitis continua':ab,ti<br>#3 #1 OR #2<br>#4 'tumor necrosis factor inhibitor'/exp<br>#5 'anti tnf agent':ab,ti OR 'anti tnf alpha agent':ab,ti OR 'anti tumor necrosis factor agent':ab,ti OR 'anti tumour necrosis factor agent':ab,ti OR 'tnf alpha inhibitor':ab,ti OR 'tnf inhibitor':ab,ti OR 'tumor necrosis factor alpha inhibitor':ab,ti OR 'tumor necrosis factor inhibitors':ab,ti OR 'tumour necrosis factor alpha inhibitor':ab,ti OR 'tumour necrosis factor inhibitor':ab,ti OR 'tumor necrosis factor inhibitor':ab,ti OR 'etanercept':ab,ti OR 'enbrel':ab,ti OR 'erelzi':ab,ti OR 'infliximab':ab,ti OR 'remicade':ab,ti OR 'adalimumab':ab,ti OR 'humira':ab,ti OR 'amjevita':ab,ti OR 'certolizumab':ab,ti OR 'cimzia':ab,ti OR 'golimumab':ab,ti OR 'simponi':ab,ti<br>#6 'interleukin 17'/exp<br>#7 'ctla 8':ab,ti OR 'ctla8':ab,ti OR 'cytotoxic t lymphocyte antigen 8':ab,ti OR 'cytotoxic t lymphocyte associated antigen 8':ab,ti OR 'cytotoxic t lymphocyte associated protein 8':ab,ti OR 'cytotoxic t lymphocyte protein 8':ab,ti OR 'il 17a':ab,ti OR 'il-17':ab,ti OR 'interleukin 17a':ab,ti OR 'interleukin-17':ab,ti OR 'interleukin 17':ab,ti OR bimekizumab:ab,ti OR bimzelx:ab,ti OR ixekizumab:ab,ti OR taltz:ab,ti OR brodalumab:ab,ti OR siliq:ab,ti OR secukinumab:ab,ti OR cosentyx:ab,ti<br>#8 'interleukin 23'/exp OR 'interleukin 12'/exp<br>#9 'il 23':ab,ti OR 'interleukin-23':ab,ti OR 'interleukin 23':ab,ti OR 'clmf':ab,ti OR 'cytotoxic lymphocyte maturation factor':ab,ti OR 'il 12':ab,ti OR 'il-12':ab,ti OR 'interleukin-12':ab,ti OR 'natural killer cell stimulatory factor':ab,ti OR 'nksf':ab,ti OR 'interleukin 12':ab,ti OR ustekinumab:ab,ti OR stelara:ab,ti OR guselkumab:ab,ti OR tremfya:ab,ti OR risankizumab:ab,ti OR skyrizi:ab,ti OR briakinumab:ab,ti OR siliq:ab,ti<br>#10'spesolimab'/exp<br>#11'bi 655 130':ab,ti OR 'bi655130':ab,ti OR 'spesolimab sbzo':ab,ti OR 'spesolimab-sbzo':ab,ti OR 'spevigo':ab,ti OR 'spesolimab':ab,ti<br>#12'janus kinase inhibitor'/exp<br>#13'jak inhibitor':ab,ti OR 'janus kinase inhibitors':ab,ti OR 'janus tyrosine kinase inhibitor':ab,ti OR 'janus kinase inhibitor':ab,ti OR tofacitinib:ab,ti OR xeljanz:ab,ti OR ruxolitinib:ab,ti OR baricitinib:ab,ti OR 'bms 986 165':ab,ti OR peficitinib:ab,ti<br>#14'phosphodiesterase iv inhibitor'/exp<br>#15'pde 4 inhibitor':ab,ti OR 'pde 4 inhibitors':ab,ti OR 'pde iv inhibitor':ab,ti OR 'pde iv inhibitors':ab,ti OR 'pde type 4 inhibitor':ab,ti OR 'pde type 4 inhibitors':ab,ti OR 'pde type iv inhibitor':ab,ti OR 'pde type iv inhibitors':ab,ti OR 'pde4 inhibitor':ab,ti OR 'pde4 inhibitors':ab,ti OR 'phosphodiesterase 4 inhibitor':ab,ti OR 'phosphodiesterase 4 inhibitors':ab,ti OR 'phosphodiesterase iv inhibitors':ab,ti OR 'phosphodiesterase type 4 inhibitor':ab,ti OR 'phosphodiesterase type 4 inhibitors':ab,ti OR 'phosphodiesterase type iv inhibitor':ab,ti OR 'phosphodiesterase type iv inhibitors':ab,ti OR 'phosphodiesterase iv inhibitor':ab,ti OR apremilast:ab,ti OR aprezo:ab,ti<br>#16#4 OR #5 OR #6 OR #7 OR #8 OR #9 OR #10 OR #11 OR #12 OR #13 OR #14 OR #15<br>#17#3 AND #16

Supplementary Table 2 (available online) shows the study characteristics, patient characteristics, acrodermatitis continua of Hallopeau (ACH) information, treatment characteristics, and outcomes of all studies included.

Supplementary Table 4 (available online) shows the study characteristics, patient characteristics, ACH information, treatment characteristics, and outcomes of 11 case series and cohort studies.

Supplementary Table 3 **Summary of patients and targeted treatments across all studies included**

Characteristics	*n*	%
Total number of patients	134	100
Sex		
Female	42	54.5
Male	35	45.5
Not reported	57	—
Age (years, mean [SD])	44.0 (22.9)	
Disease duration (years, mean [SD])	6.7 (7.5)	
Received targeted treatments before	26	19.4
Targeted treatment courses	209	100
IL-17 inhibitors	43	20.6
Ixekizumab	16	7.7
Secukinumab	22	10.5
Brodalumab	4	1.9
Bimekizumab	1	0.5
IL-12/23 inhibitors	38	18.2
Ustekinumab	24	11.5
Guselkumab	10	4.8
Risankizumab	3	1.4
Tildrakizumab	1	0.5
TNF-α inhibitors	107	51.2
Etanercept	29	13.9
Infliximab	25	12.0
Adalimumab	51	24.4
Certolizumab	2	0.9
IL-1 inhibitors (anakinra)	3	1.4
IL-36 inhibitors (spesolimab)	1	0.5
Anti-CD11a antibodies (efalizumab)	3	1.4
PDE4 inhibitors (apremilast)	12	5.7
JAK1/2 inhibitors (baricitinib)	2	1.0

Abbreviations: SD, standard deviation; IL, interleukin; TNF, tumor necrosis factor; PDE, phosphodiesterase; JAK, Janus kinase.

Supplementary Table 5 **Summary of patient information as well as disease and treatment characteristics across 11 case series and cohort studies**

Characteristics	*n*	%
Patient details		
Family history		
Yes	2	2.5
No	23	29.1
NR	54	68.4
Comorbidity		
Psoriasis vulgaris	4	5.1
Psoriatic arthritis	4	5.1
Inverse psoriasis	1	1.3
Palmoplantar pustulosis	1	1.3
Cardiovascular disease	4	5.1
Psychiatric disease	2	2.5
Alcohol use disorder	1	1.3
Smoking	6	7.6
None	3	3.8
NR	59	74.7
IL36RN genetic mutation		
c.115+6T>C	8	10.1
c.227C>T	2	2.5
No	2	2.5
NR	67	84.8
Clinical features		
Extent of lesions		
Involvement of fingers and toes	13	16.5
Finger involvement only	13	16.5
Toe involvement only	0	0
NR	53	67.1
Enthesitis		
Yes	10	12.7
No	12	15.2
NR	57	72.1
Bone erosions		
Yes	5	6.3
No	12	15.2
NR	62	78.5
Prior failed treatments		
Traditional treatment		
Corticosteroids	8	10.1
Cyclosporine A	10	12.7
Methotrexate	12	15.2
Fumaric acid esters	2	2.5
Acitretin	17	21.5
Antibiotics	3	3.8
Phototherapy	2	2.5
Biologics	12	15.2
Small molecule drug	4	5.1
NR	52	65.8

Abbreviation: NR, not reported.

Supplementary Table 6 **Summary of targeted therapy outcomes in patients with acrodermatitis continua of Hallopeau**

Outcomes	IL-17 inhibitors			IL-12/23 inhibitors			TNF-α inhibitors			PDE4 inhibitors	JAK1/2 inhibitors	IL-1 inhibitors	CD11a inhibitors
	Ixekizumab (*n*=9)	Secukinumab (*n*=13)	Brodalumab (*n*=1)	Ustekinumab (*n*=13)	Guselkumab (*n*=5)	Risankizumab (*n*=1)	Etanercept (*n*=16)	Infliximab (*n*=12)	Adalimumab (*n*=31)	Apremilast (*n*=2)	Baricitinib (*n*=1)	Anakinra (*n*=1)	Efalizumab (*n*=1)
CR (*n*[%])[1]	2 (22.2)	3 (23.1)	1 (100)	4 (30.8)	5 (100)	1 (100)	3 (18.8)	2 (16.7)	21 (67.6)	0 (0)	0 (0)	0 (0)	0 (0)
CR (*n*[%])[2]		M, 1 (14.3)<br>F, 2 (33.3)<br>NR, 3 (30.0)			M, 2 (50.0)<br>F, 3 (75.0)<br>NR, 5 (45.5)			M, 5 (45.5)<br>F, 10 (66.7)<br>NR, 11 (32.4)					
CR time (weeks)	30	NR	12	40[a]	16[b]	24	NR	NR	14.57[c]	NA	NA	NA	NA
PR	1	2	0	2	0	0	2	5	4	0	0	0	0
NO	6	8	0	7	0	0	11	5	6	2	1	1	1
Adverse events (*n*)	Yes, 0<br>No, 2<br>NR, 7	Yes, 2<br>No, 10<br>NR, 6	Yes, 0<br>No, 0<br>NR, 1	Yes, 2<br>No, 4<br>NR, 7	Yes, 0<br>No, 5<br>NR, 0	Yes, 0<br>No, 1<br>NR, 0	Yes, 1<br>No, 4<br>NR, 11	Yes, 5<br>No, 0<br>NR, 7	Yes, 3<br>No, 18<br>NR, 10	NA	NA	NA	NA
AEI (%)	0	15.4	0	15.4	0	0	6.3	41.7	9.7	NA	NA	NA	NA

[1]Complete response (CR) rate based on the total number of patients in each subgroups.
[2]Complete response rate based on the reported sex information of patients in each groups.
[a]The CR time recorded for one of the four cases achieved CR with Ustekinumab treatment.
[b]The CR time recorded for three of the five cases achieved CR with Guselkumab treatment.
[c]The CR time recorded for 14 of the 21 cases achieved CR with Adalimumab treatment.
Abbreviations: F, female; M, male; NR, not reported; NA, not available; CR, complete response; PR, partial response; NO, no resolution; AEI, adverse events incidence.
